# *Arabica*-coffee and *teobroma*-cocoa agro-industrial waste biosorbents, for Pb(II) removal in aqueous solutions

**DOI:** 10.1007/s11356-022-22233-3

**Published:** 2022-08-08

**Authors:** Carmencita Lavado-Meza, Leonel De la Cruz-Cerrón, Gregorio Cisneros-Santos, Alex H. De la Cruz, Julio Angeles-Suazo, Juan Z. Dávalos-Prado

**Affiliations:** 1 Escuela Profesional de Ingeniería Ambiental, Universidad Nacional Intercultural de la Selva Central Juan Santos Atahualpa, Av. Perú 612, Chanchamayo, Perú; 2 Facultad de Ingeniería de Sistemas, Universidad Nacional del Centro del Perú, Av. Mariscal Castilla No 3909, Huancayo, Perú; 3 Escuela Profesional de Administración de Negocios Internacionales, Universidad Nacional Intercultural de la Selva Central Juan Santos Atahualpa, Av. Peru 612, Chanchamayo, Perú; 4 Facultad de Ingeniería y Arquitectura, Universidad Peruana Unión, Carretera Central Km 19.5, Ñaña, Perú; 5 Facultad de Ingeniería Industrial, Universidad Tecnológica del Perú, Av. Arequipa 265, Lima, Perú; 6 Instituto de Química Física Rocasolano, CSIC, Madrid, Spain

**Keywords:** Coffee residues, Cocoa residues, Heavy metals, Biosorption, Agricultural waste, Pb(II) removal, Langmuir isotherms

## Abstract

Agro-industrial waste biosorbents of *arabica*–coffee (WCA) and *theobroma*–cocoa (WCT) have been characterized and tested to remove Pb(II) from aqueous media. The maximum adsorption capacity of WCA and WCT (q_max_ = 158.7 and 123.5 mg·g^−1^, respectively) is comparable or even higher than for several other similar agro-industrial waste biosorbents reported in the literature. Structural and morphological characterization were performed by infrared spectrometry with Fourier transform (FT-IR), scanning electron microscopy/energy-dispersive X-ray spectroscopy (SEM/EDS), and charge measurements at the zero point charge (pH_PZC_). Both biosorbents, WCA and WCT, show cracked surfaces with heterogeneous plates which ones include functional adsorption groups such as OH, C = O and C-O-C. Optimal Pb(II) adsorption occurs for a pH between 4 and 5 at [WCA] and [WCT] dose concentrations of 2 g·L^−1^. We found that the adsorption process follows pseudo-second order kinetics with a rapid growth rate (almost six times larger for WCA than for WCT), basically controlled by the chemisorption process. The regeneration of both biosorbents was carried out in an eluent of 0.1M HNO_3_ and they can be efficiently reused up to 5 times.

## Introduction

At present, anthropogenic contamination of aquatic ecosystems is one of the major emerging problems with global implications (Häder et al. 2020; Salazar-Pinto et al. 2021). Aquatic ecosystems can be polluted through different sources, such as domestic effluents, industrial and extraction discharges related to mining, and oil refineries. Inorganic pollutants include metals and their derivatives from the corrosion of metal structures, mining extraction residues, and industrial discharges such as electroplating and battery manufacturing (Negm et al. 2018). Heavy metals are especially dangerous due to their toxicity, persistence, and ability to bioaccumulate in the food chain of living beings (Flores-Trujillo et al. 2021). This is the case of Pb(II) that when ingested produces severe disorders in the body, particularly in the nervous system and fertility (Lee et al. 2019; Lentini et al. 2019; Shooto et al. 2020). Therefore, the effective elimination of Pb from wastewater and in general, the investigation of heavy metal biosorption processes on biological surfaces or biosorbent materials (Beni and Esmaeili 2020) is highly current. We highlight the use of various agricultural by-products as “cheap” biosorbents to remove Pb from aqueous solutions, such as olive tree pruning (Calero et al. 2013), taro (Saha et al. 2017), prickly pear stalk (Lavado-Meza et al. 2020), sugar cane bagasse (Tejada-Tovar et al. 2020), spent coffee grounds (Ayucitra et al. 2017; Chwastowski et al. 2020) coffee grounds (Minamisawa et al. 2004; Gomez-Gonzalez et al. 2016), coffee husk (Oliveira et al. 2008), endocarp waste coffee (Gómez-Aguilar et al. 2021; Mariana et al. 2021), and untreated cocoa shells (Meunier et al. 2003; Obike et al. 2018). The use of these materials is attractive due to their availability and the low costs involved in treating contaminated water.

In recent years, coffee and cocoa production in Peru has increased to 135.9 and 218 Ktons/year, respectively. Of the total national production of Peru, the production in Junín (Central-Selva region) represents 35 and 12% for coffee and cocoa respectively. In the coffee agribusiness, its cherries are processed generating large amounts of residues from pulping and washing, in such a way that a ton of coffee can produce 600 kg of residues (Anastopoulos et al. 2017). On the other hand, in the cocoa processing chain, the main residue is the shell, which represents between 70 and 80% of the total fruit (Vásquez et al. 2019). The large-scale generation of residual biomass represents a concern of cocoa and coffee producing countries since if they are not properly processed and/or treated, they can generate environmental problems in both aquifer and soil systems (Anastopoulos et al. 2017). In this context, the goals of this work are the following:

- Characterize the agro-industrial waste biosorbents, arabica coffee (WCA) and theobroma cocoa (WCT), regarding the morphology and surface structure.
- Evaluate the parameters that affect the sorption of the WCA and WCT.
- Kinetic study. Mechanisms of sorption processes;
- Study the regeneration process of biosorbents (WCA, WCT) and the feasibility of re-use through different desorbent agents.

## Methods

### Preparation of biosorbents

*Arabica*-coffee (WCA) and *theobroma*-cocoa (WCT) agro-industrial waste were collected from Satipo and Chanchamayo provinces located at Junin, Perú. Both samples were previously washed with water then rinsed with distilled water, dried at 70 ^o^C for 48 h. After that, the dried absorbents were ground and sieved using a 70-mesh. All chemicals reagents used in this work were of analytical grade

### Characterizations of the biosorbents

The study of the Point of Zero Charge (PZC) was evaluated according to the procedures described by do Nascimento et al. (2019). It has been prepared a mixture of 0.05 mg of biosorbent with 50 mL of aqueous solutions under different initial pHs (pH_0_) ranging from 1 to 8. The acid dilutions were prepared from 1M HCl solution, while the basic dilutions from 1M NaOH. After 24 h of equilibrium, the final pHs (pH_f_) were determined.

Fourier transform infra-red spectrophotometer (FTIR, SHIMADZU- 8700) was used to characterize the functional groups present on the surface of biosorbents. The wavelength was set to 4000 to 500 cm^-1^.

Morphological and elemental analysis on the surface of WCA and WCT biosorbents were performed by scanning electron microscopy (SEM) coupled with EDS (energy-dispersive X-rays spectroscopy) (Hitachi SU8230 model).

### Adsorption experiments

The effect of experimental parameters including pH, biosorbent dosage, contact time, initial concentration, kinetic and isothermal models on the adsorption of Pb(II) on the WCA and WCT biosorbents has been studied. Between 0.025 and 0.2 g amount of each biosorbent was added to 25 mL of Pb(NO_3_)_2_ solution, with a varying [Pb(II)] concentrations between 26.9 and 196.4 mg·L^−1^. These solutions had been adjusted to pH in the range of 2.0–5.0 by adding 0.1M HNO_3_ or 0.1M NaOH. The suspension was stirred to 150 rpm for the time period of 0–180 min. The temperature was kept at room temperature. The amount of adsorption of Pb(II) (q_*e*_) onto the biosorbents was determined by measuring the concentration of lead in the resulting filtrate with an SHIMADZU-AAS 6800 instrument Atomic Absorption Spectrophotometer (AAS at 283.3 nm). Both, adsorption capacity q_*e*_ (in mg·g^−1^) and removal efficiency (%R), were calculated by using Eqs. (1) and (2), respectively (Morosanu et al. 2017):

1 $${\mathrm q}_{\mathrm e}\frac{{\mathrm C}_0-{\mathrm C}_{\mathrm e}}{\mathrm m}\times\mathrm V$$

2 $$\%\mathrm R=\frac{{\mathrm C}_0-{\mathrm C}_{\mathrm e}}{{\mathrm C}_0}\times100$$

where *C*_0_ and *C*_e_ (in mg·L^−1^) are the initial and equilibrium final Pb(II) concentrations, respectively; V(in L) is the volume of solution and m (in g) is the biosorbent mass. The adsorption experiments were repeated for 3 times and the average values were used to report.

The kinetic data were evaluated with the pseudo first, Largergren (pseudo-second order) and the intraparticle-diffusion models (Shooto et al. 2020). The experimental data of the adsorption isotherms were correlated to the Langmuir (Eq. (3), Langmuir 1916; Foo and Hameed 2010) and Freundlich (Eq. (4), Freundlich 1906) models (Tran et al. 2016):

3 $$\frac{C_e}{q_e}=\frac{1}{bq_{\mathrm{max}}}+\frac{C_e}{q_{\mathrm{max}}}$$

where b and q_max_ are Langmuir constants related to, respectively: the affinity between sorbent-sorbate and maximum biosorption capacity

4 $${\mathrm{lnq}}_{\mathrm e}={\mathrm{lnK}}_{\mathrm F}+\frac1{\mathrm n}{\mathrm{lnC}}_{\mathrm e}$$

where K_F_⋅and n are related to, respectively: equilibrium and the affinity between sorbent and sorbat^n^

### Desorption experiments

The desorption process was carried out using five types of eluent acids 0.1M (HCl, HNO_3_, H_2_SO_4_, CH_3_COOH and NaOH). One hundred milligrams of each biosorbent previously loaded with Pb(II), from the mixture with 50 mL of lead solution ([Pb(II)] = C_0_ = 75.8 mg·L^−1^) then filtered and dried was subjected to the desorption process by adding 50 mL of each aforementioned eluent and then stirred at 120 rpm for 2h. After that, the biosorbents were washed with distilled water, dried, and re-used again. The adsorption/desorption operation was repeated up to 6 times. The [Pb(II)] concentration adsorbed and desorbed was analyzed by atomic absorption spectroscopy (before described).

The desorption efficiency (%Des) of the biosorbents studied was calculated using the following expression (Nayak and Pal 2017):

5 $$\%\mathrm D=\frac{\mathrm{Pb}(\mathrm{II})\mathrm{desorbed}}{\mathrm{Pb}(\mathrm{II})\mathrm{sorbed}}\times100$$

## Results and discussion

### Biosorbent properties, pH effect

**Fig. 1:**
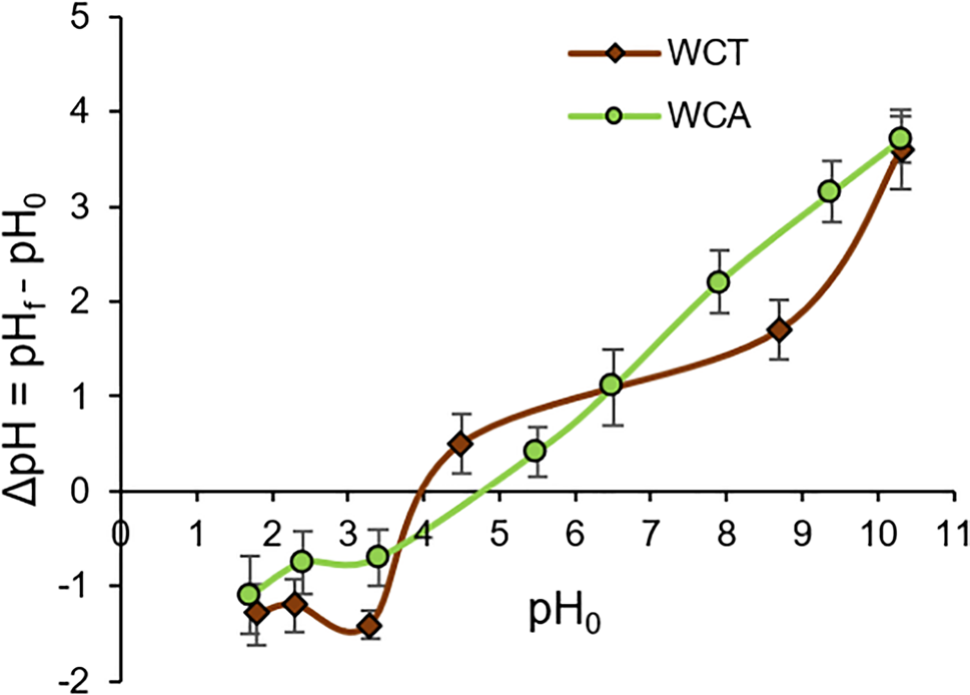
Determination of pH_PZC_ values for WCA (green) and WCT (red) biosorbents

The zero charge point pH values (pH_PZC_) were obtained from the representation of ΔpH (= pH_0_ - pH_f_) *vs* initial pH_0_ values (see Fig. 1). The two curves belonging to the WCA and WCT intersect the pH_0_ axis at, respectively pH_PZC_ = 4.8 and 3.9. It indicates that the surfaces of the WCA and WCT are positively charged at, respectively pH < 4.8 and pH < 3.9. For pH > pH_PZC,_ the biosorbent surface is negatively charged (Moghazy et al. 2019; Morosanu et al. 2017) favoring electrostatic attraction of Pb(II) at higher pH values.

**Fig. 2:**
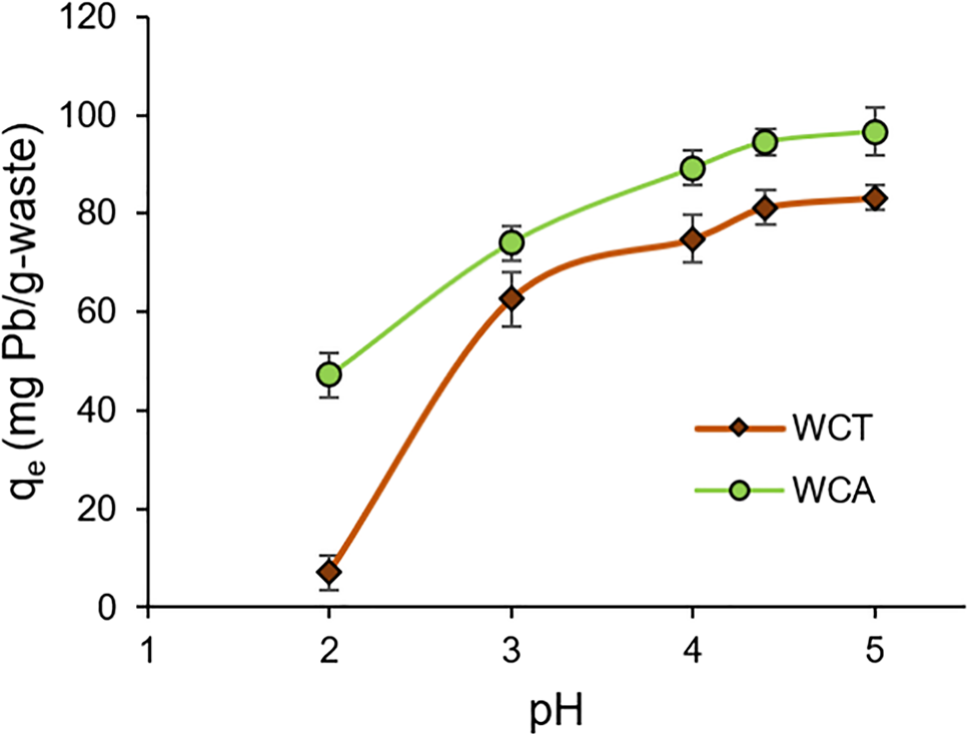
Influence of pH on the Pb(II) removal capacity q_e_. Experimental conditions: T = 20 ^o^C, sorption time t_sp_ = 120 min, biosorbent mass m = 0.05 g (S/L solid/liquid ratio= 2 g⋅L^-1^), C_0_ = 99.7 mg⋅L^-1^

It is important to point out the pH influence on the capacity of metal-ion sorption, since it determines the surface properties of the adsorbent in terms of surface charges, ionization, and dissociation degree of functional groups on the adsorbent active-sites (Ezeonuegbu et al. 2021). Figure 2 shows the influence of pH of the Pb(II) sorption capacity q_e_, on WCT and WCA biosorbents. So, for acidic pHs, we can note how the sorption capacity q_e_, for both biosorbents, increases until reaching a maximum close to pH = 5 (see Fig. 2). This behavior is related to the competition of H_3_O^+^ and Pb(II) ions to occupy biosorbent active-sites. Thus, for pH < pH_PZC_, the H_3_O^+^ concentration is high and most of the biosorbent active-sites can be occupied by these ions. In such situation, the adsorbent repels the Pb(II) ions reducing its adsorption capacity. The opposite case is for pH > pH_PZC_, since the bioabsorbent surface is negative and increases its Pb(II) adsorption capacity q_e_. In our case, we found an increasing Pb removal up to pH 5, however already for small increments of this value, we observed a rapid precipitation of Pb. The literature report suppression of Pb(II) bioadsorption above pH 5-6 (Elkhaleefa et al. 2021; Ali et al. 2019), which would be -according to Pb-Pourbaix diagram- due Pb(II) precipitation to Pb(OH)_2_.

Our results are comparable to those reported in the literature for optimal Pb(II) adsorption conditions (pH range between 4 and 6) determined for organic residues such as the carnauba palm (Oliveira et al. 2021), banana peel (Afolabi et al. 2021), tomato residues (Heraldy et al. 2018), and particularly coffee husk and pulp residues (Zaragoza et al. 2011) where at pH = 4.25 almost 100% of Pb is removed from solution.

**Fig. 3:**
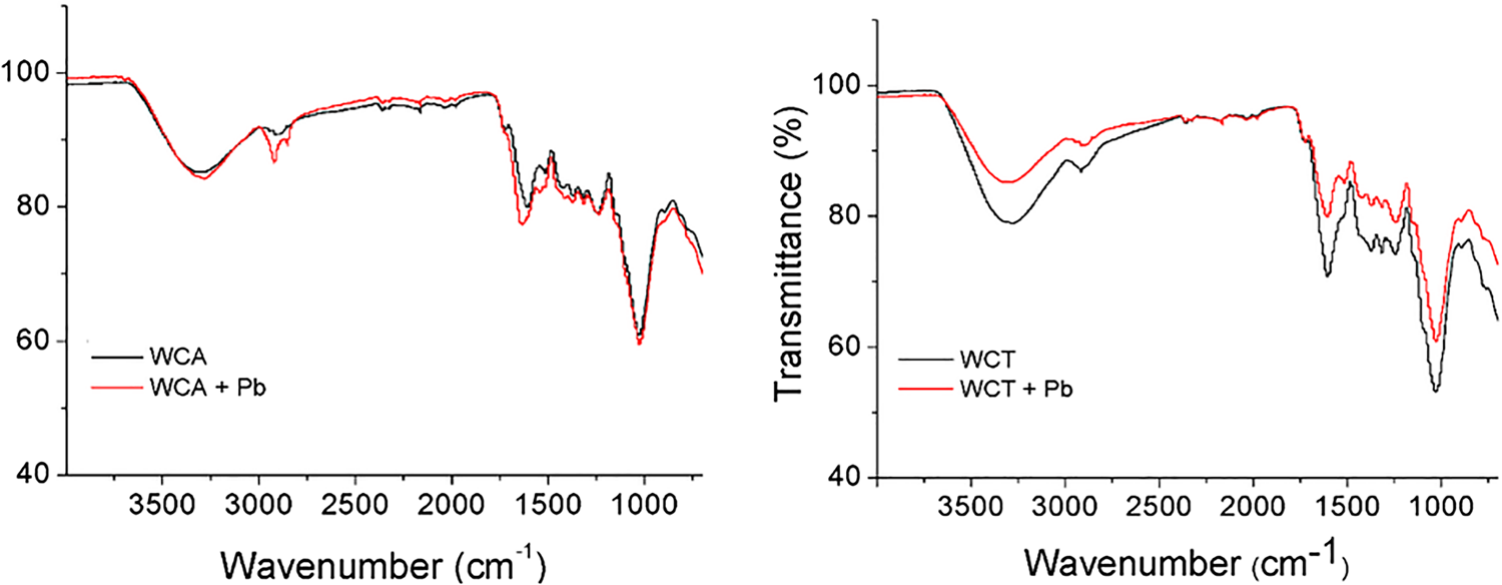
Typical FTIR spectra before (black) and after (red) of Pb(II) sorption by WCA (left) and WCT (right). pH = 4.5, C_0_ = 127.3 mg⋅L^-1^, S/L = 2 g.L^-1^

FTIR spectral analysis was not only used to identify functional groups present on the surface of WCA and WCT biosorbents but also to investigate interactions between functional groups and Pb(II) ions. Figure 3 shows typical FTIR spectra of WCA and WCT, with similar absorption peaks or spectral bands, before and after Pb(II) sorption. The FTIR spectra of the clean samples show band positions (in parentheses for WCT) at 1/ 3276.6 (3291.4) cm^-1^, assignable to typical -OH bond stretching vibrations in samples such as cellulose and lignin (Flores-Trujillo et al. 2021; Taşar et al. 2014); 2/ 2910.2 (2914.6) and 1236.1 (1249.0) cm^-1^ which would be related to the symmetric stretching of the C-H bonds typical in lignocellulosic samples (Heraldy et al. 2018); 3/ 1627.9 (1600.9) cm^-1^ assignable to the asymmetric stretching of the double bond of C = O carbonyl groups (Barka et al. 2013); and 4/ 1373.4 (1366.3) cm^-1^, assignable to the stretching C-OH and C = O groups of carboxylates (Barka et al. 2013); 5/ 1026.1 (1026.1) cm^-1^, characteristic of C-O-C stretching in polysaccharides (Morosanu et al. 2017);

FTIR spectra after contact with the Pb(II) solution show changes in the intensity and position of some adsorption peaks. Thus, for both biosorbents, the positions of the bands 1/ and 3/ are significantly displaced, respect to the clean sample values, at Δ_1_ = 16.4 (12.93) and Δ_3_ = -24.6 (11.8) cm^-1^. For WCT also the band 5/ is significantly displacement at Δ_5_ = (5.7) cm^-1^. These results indicate that the O-H, C-O and C-O-C groups would be involved in the biosorption of Pb(II) (Morosanu et al. 2017). A similar behavior was reported by Barka et al., (2013) in the removal of Pb and Cd by cladodes of prickly pear.

**Fig. 4:**
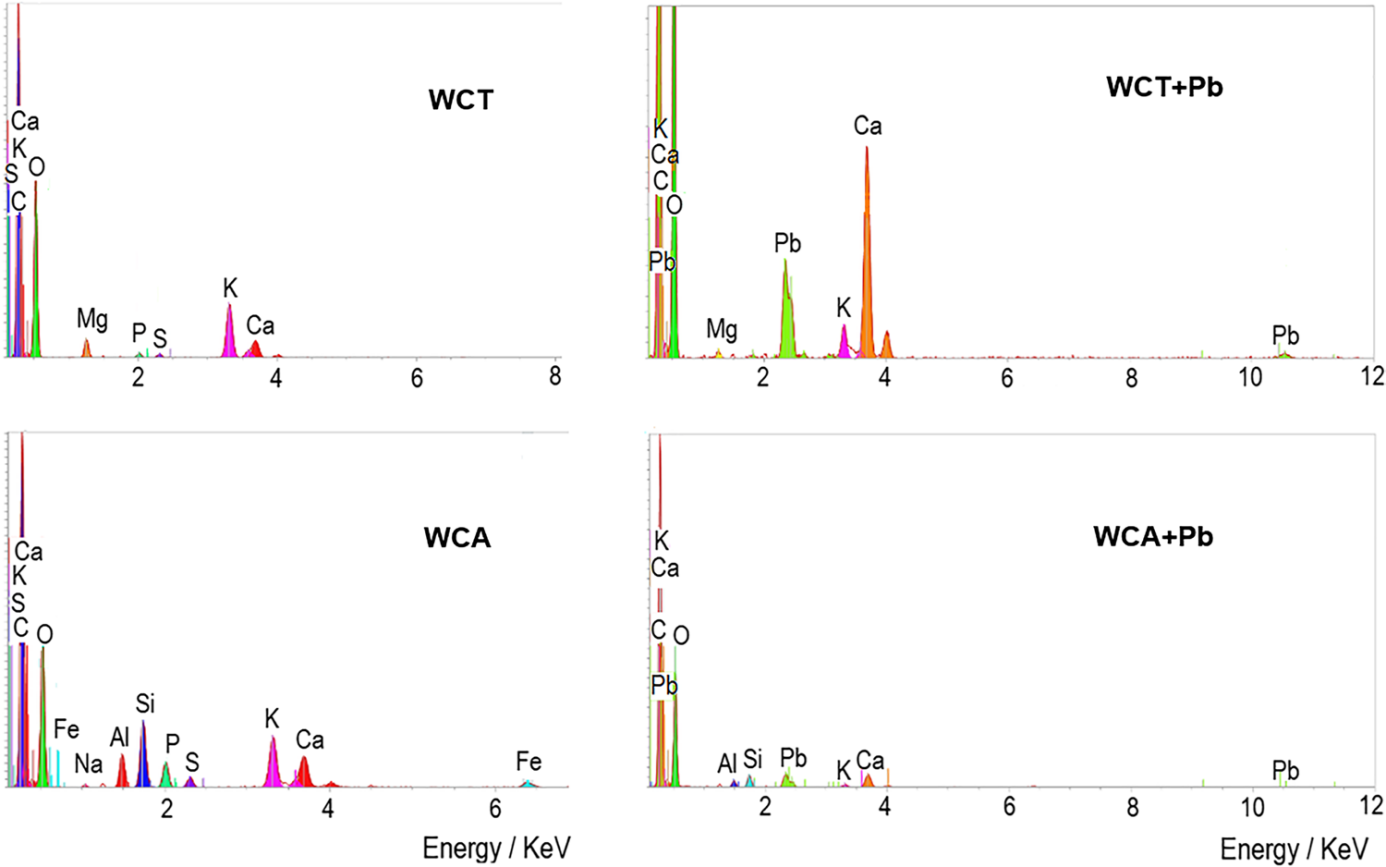
Typical EDS spectra of WCT and WCA. Before (left) and after (right) of Pb(II) sorption. pH = 4.5, C_0_ = 127.3 mg⋅L^-1^, S/L= 2 g.L^-1^

Typical EDS spectra of WCT and WCA are shown in Fig. 4, before and after the Pb(II) sorption processes. Both clean biosorbents (Fig. 4, left) show the presence of common elements such as C, O, P, S, K, and Ca. WCT includes also to Mg and WCA to Fe, Al, Na, and Si.

After sorption process (Fig. 4, right) clearly can be identified Pb peaks. In this regard, El-Naggar et al. (2018) reported intense Pb peaks in EDS spectra of *Gelidium amansii* biomass after treatments with Pb solutions.

**Fig. 5:**
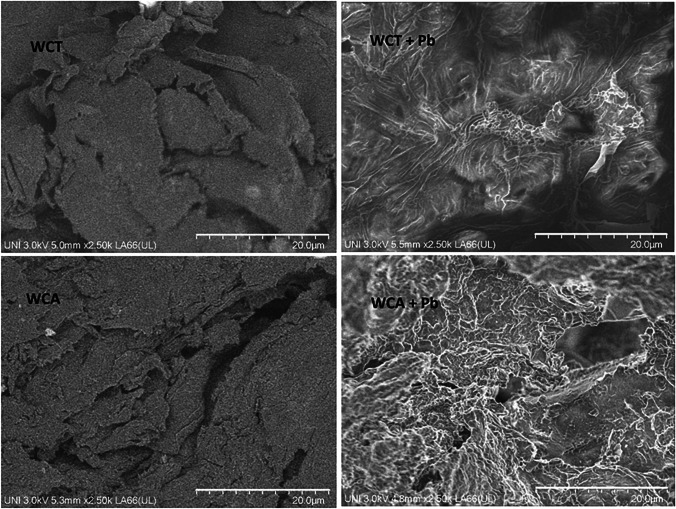
Typical SEM morphology of WCA and WCT, before (left) and after (right) of Pb(II) sorption. pH = 4.5, C_0_ = 127.3 mg⋅L^-1^, S/L = 2 g L^-1^

SEM typical images of the WCT and WCA before and after the sorption of Pb(II) are shown in Fig. 5. We can see that there are significant morphological differences of the surfaces of both biosorbents between before and after Pb(II) sorption. Thus, the clean biosorbents show cracked surfaces with heterogeneous plates that would facilitate the sorption of Pb(II), while both WCT and WCA after sorption are more homogeneous. Similar morphologies are observed in the works reported by Saha et al. (2017) and Fomina and Gadd (2014) after the removal of Pb(II) using Tara and Colza residues respectively.

### Sorption tests

The removal percentage %R of Pb(II), in solution (C_0_ = 26.96 mg⋅L^-1^), was evaluated as a function of biosorbent mass, at room temperature, pH = 4.5 and sorption time t_sp_ = 120 min.

**Fig. 6:**
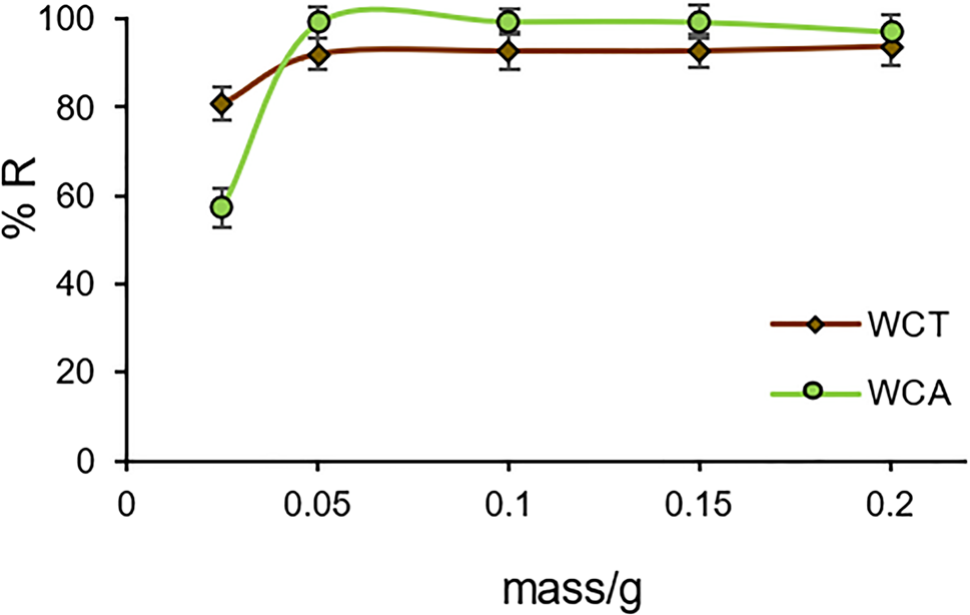
Pb(II) removal percentage (%R) as a function of the biosorbent mass. C_0_ = 26.96 mg⋅L^-1^ at pH = 4.5, sorption time t_sp_ = 120 min

Both WCT and WCA biosorbents reach the maximum removal percentage approximately %R_max_ = 92 and 99, respectively, for a mass m = 0.05 g (see Fig. 6), which corresponds to biosorbent-solid/liquid ratio of S/L= 2 g⋅L^-1^. %R_max_ values are related to the structure of the biosorbent that determines the distribution and sites number of the adsorbed species (Negm et al. 2018; Blázquez et al. 2014).

**Fig. 7:**
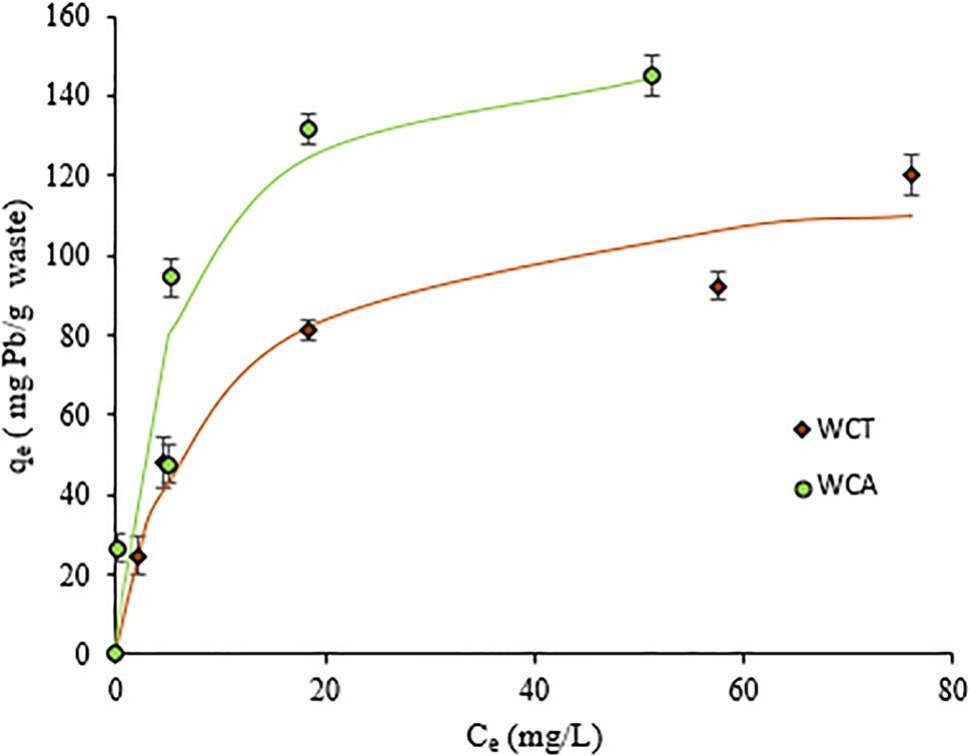
WCT and WCA adsorption isotherms. Conditions: pH = 4.5, T = 20 ^o^C, biosorbent mass m = 0.05 g (S/L= 2g.L^-1^), adsorption time t_ad_ = 120 min. Continue curves correspond to nonlinear fits (Langmuir model)

The adsorption isotherms were studied in a range of initial [Pb(II)] concentrations C_0_ between 26.98 and 196.4 mg·L^−1^, pH = 4.5, T = 20 ^o^C and t_sp_ = 120 min. The results are shown in Fig. 7, depicting the adsorption capacity q_e_ (mg of Pb(II)/g-mass of biosorbent) vs. the concentration of Pb(II) in equilibrium, C_e_. The experimental data were fitted to Langmuir and Freundlich models for both, nonlinear and linear correlations. The concave shape of the reported isotherms (Fig. 7) indicates the significant affinity of WCT and WCA for Pb(II) sorption (Taşar et al. 2014). For linear correlations, the experimental data of the isotherms were regrouped as [C_e_/q_e_ *vs* C_e_] and [log(q_e_) *vs* log(C_e_)] to be adjusted by applying, respectively, the Langmuir and Freundlich models (Fomina and Gadd 2014). The Langmuir model assumes a solute sorption in monolayers with a homogeneous sorption energy (Beni and Esmaeili 2020), while the Freundlich model assumes multilayer sorption, with heterogeneous sorption energies (Srivastava et al. 2015). The adjustment parameters with both models are shown in Table 1.

**Table 1:** Adjustment parameters of Pb(II) sorption isotherms, in equilibrium, with Langmuir and Freundlich models

Biosorbent	Langmuir parameters			Freundlich parameters		
	q_max_/ mg⋅g^-1^	K_L_ / Lmg^-1^	R^2^	K_F_ / mg g^-1^L^(1/n)^mg^-(1/n)^	1/n	R^2^
WCT	123.5 ± 13.5 (119.0 ± 10.8)	0.11 ± 0.07 (0.13 ± 0.05)	0.97 (0.92)	41.5 ± 7.8 (46.0 ± 13.8)	0.34 ± 0.07 (0.31±0.09)	0.87 (0.80)
WCA	158.7 ± 17.4 (166.9 ± 30.4)	0.20 ± 0.12 (0.16 ± 0.09)	0.97 (0.81)	22.2 ± 4.3 (26.3 ± 6.7)	0.39 ±0.06 (0.34±0.07)	0.93 (0.90)

Between brackets, results obtained from nonlinear fits

We can see that isotherm adsorptions are fitted with higher R^2^ for linear than for nonlinear correlations (Table 3) and better with Langmuir model (R^2^ = 0.97) than the Freundlich model (R^2^ = 0.87 and 0.93). From the first model, we can derive low K_L_ values (see Table 1) with the maximum sorption capacity q_max_ equal to 158.7 and 123.5 mg Pb(II)/g-biosorbent, for WCA and WCT, respectively. These values indicate high affinity of Pb(II) sorption (Barquilha et al. 2019). Despite the worst adjustments obtained with the Freundlich model, we do not rule out sorption processes in multilayers of a heterogeneous nature (Ou et al. 2015). The corresponding 1/n adjustment values (between 0 and 1) also indicate that the biosorption of Pb (II) on WCT and WCA is favorable under the conditions studied (Lavado-Meza et al. 2021; Reddy et al. 2010). All these results show that in reality the sorption of WCT and WCA is rather complex process (Saha et al. 2017).

q_max_ values of agro-industrial wastes similar to those studied in this work are shown in the Table 2. We can note that q_max_ of WCT and WCA are among the highest. It is interesting to mention that q_max_ of WCA is comparable to the value recently obtained by (Mariana et al. 2021) for Gayo-Coffee.

**Table 2:** Comparative table of the maximum biosorption capacity q_max_, of Pb(II) for agro-industrial wastes

Biosorbent wastes	q_max_ (mg/g)	Reference
Spent coffee grounds Coffee ground Untreated coffee residues Gayo coffee Endocarp waste coffee *Arabica*-coffee (WCA)	13.6 22.9 9.7 174.4 24.10 158.7	Chwastowski et al. 2020 Gomez-Gonzalez et al. 2016 Wu et al. 2015 Mariana et al. 2021 Gómez-Aguilar et al. 2021 This work
Cocoa pods Cocoa shells *Teobroma*-cocoa (WCT)	4.83 6.2 123.5	Obike et al. 2018 Meunier et al. 2003 This work

### Kinetic studies

**Fig. 8:**
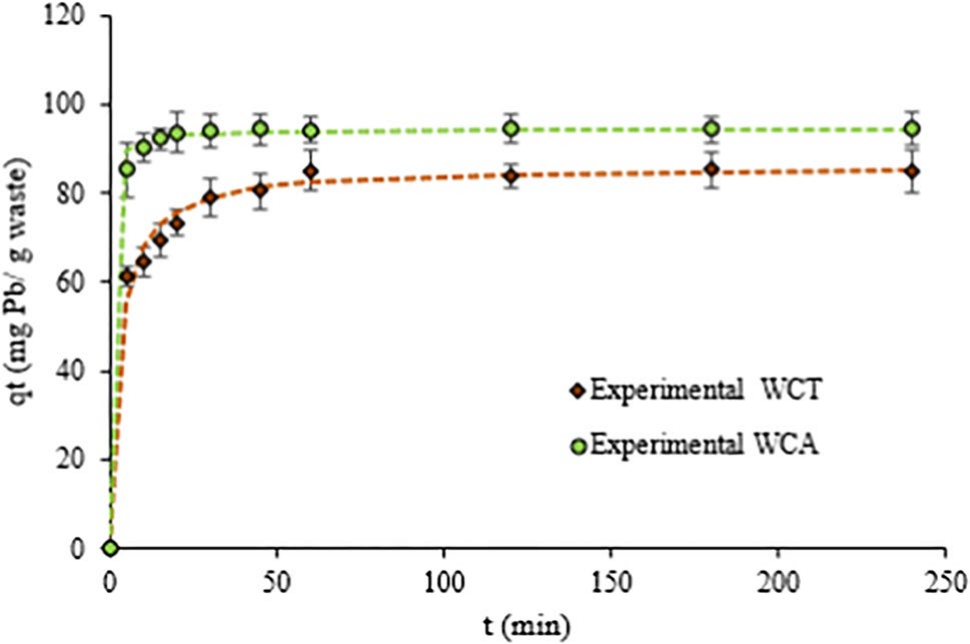
q_t_ (amount Pb(II) removed per mass unit of biosorbent) *vs* time t (min). C_0_ = 127.5 mg⋅L^-1^, pH = 4.5. Dotted lines corresponding to pseudo-2^nd^ order adjustments

Figure 8 shows the results of the kinetic tests carried out to determine the equilibrium time required for Pb(II) sorption on WCT and WCA. A rapid increase of q_t_, amount of Pb(II) removed per unit mass of biosorbent at time t, is appreciate until reaching its maximum value after approx. 60 minutes. For longer times (t > 60) practically q_t_ remains constant.

The experimental kinetic data, previously configured, were adjusted using three kinetic models of adsorption: log (q_e_-q_t_) *vs* time t (pseudo first-order model); t/q_t_ vs. time t (pseudo second-order model) and q_t_ vs. t^1/2^ (intraparticular diffusion model) (Ezeonuegbu et al. 2021). The parameters obtained after the optimized adjustments and their corresponding qualities (correlation coefficient R^2^) are reported in Table 3.

**Table 3:** Parameters for the adjustment of experimental data using kinetic models. T = 20 ^o^C, C_0_ = 99.7 mg⋅L^-1^ [Pb(II) initial concentration]

Model	Parameters	Biosorbent	
		WCT	WCA
*Pseudo* 1^st^-order	k_1_	0.02	0.07
	q_e,cal_	17.98	6.12
	R^2^	0.95	0.78
*Pseudo* 2^nd^-order	k_2_	0.006	0.035
	q_e,cal_	86.21	94.34
	h	46.7	312.5
	R^2^	0.99	1
Intra-particle diffusion	k_id I_	5.47	3.78
	k_id II_	3.29	0.16
	k_id III_	0.02	0.04

k_1_ (1⋅min^-1^): the 1^st^-order kinetic constant; q_e,cal_ (mg⋅g^-1^) calculated adsorption capacity; k_2_ (g⋅mg^-1^⋅min^-1^) rate constant adsorption, h (mg⋅g^-1^⋅min^-1^) initial adsorption rate; k_id_ (mg⋅g^-1^⋅ min^-1/2^) intraparticle diffusion rate constant

**Fig. 9:**
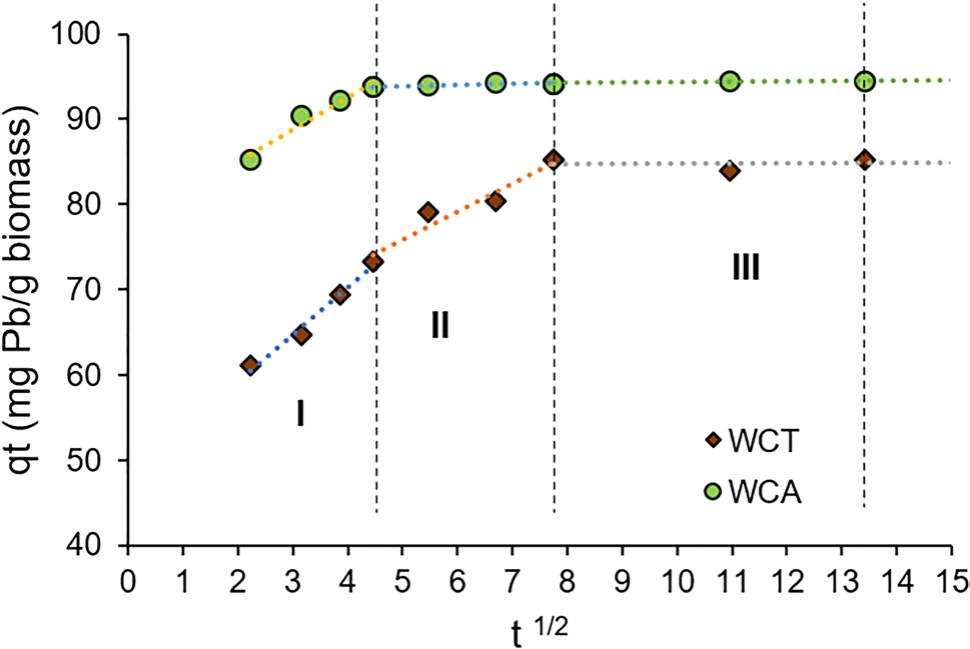
Weber–Morris plots of Pb(II) sorption on to WCT and WCA

For both biosorbents, we can note a better correlation (R^2^ ≈ 1) with pseudo-2^nd^ order than the 1^st^-order adjustment models. Accordingly, we can affirm that (i) the adsorption of Pb(II) is a chemisorption process; (ii) the calculated adsorption capacities q_e,cal_ are close to those determined experimentally; (iii) the adsorption rates (k_2_ and h) for WCA are almost six times larger than for WCT.

q_t_ vs. t^0.5^ data, for both WCA and WCT biosorbents, are represented in the Fig. 9 and fitted with the intra-particle diffusion Weber –Morris model. According to the k_id_ intra-particle diffusion rate constants, we can distinguish three parts: 1^st^ part showing a rapid growth of q_t_ at the time t (k_id,I_ > 3.7) which would indicate the rapid absorption of Pb(II) ions on the outer surface of the bioabsorbents; 2^nd^ part, a slower growth of q_t_ with t (0.16 < k_id,II_ < 3.3), which would be related to a gradual sorption process, where Pb(II) ions would enter and filling to the biosorbent pores. It would be the stage that controls the rate of diffusion towards the mesopores; finally 3^rd^ part, where q_t_ is practically invariant or constant with very low k_id,III_ values (see Table 3), it would indicate that intra-particle diffusion in micropores is the limiting step of the speed in the sorption process (Saha et al. 2017). Morosanu et al. (2017), Negm et al. (2018) and Blázquez et al. (2014) reported similar results applying intra-particular diffusion Weber–Morris model for Pb(II) sorption on, respectively, rapeseed biomass, brown-algae fungi and olive-stone.

### Desorption and regeneration of biosorbents

**Fig. 10:**
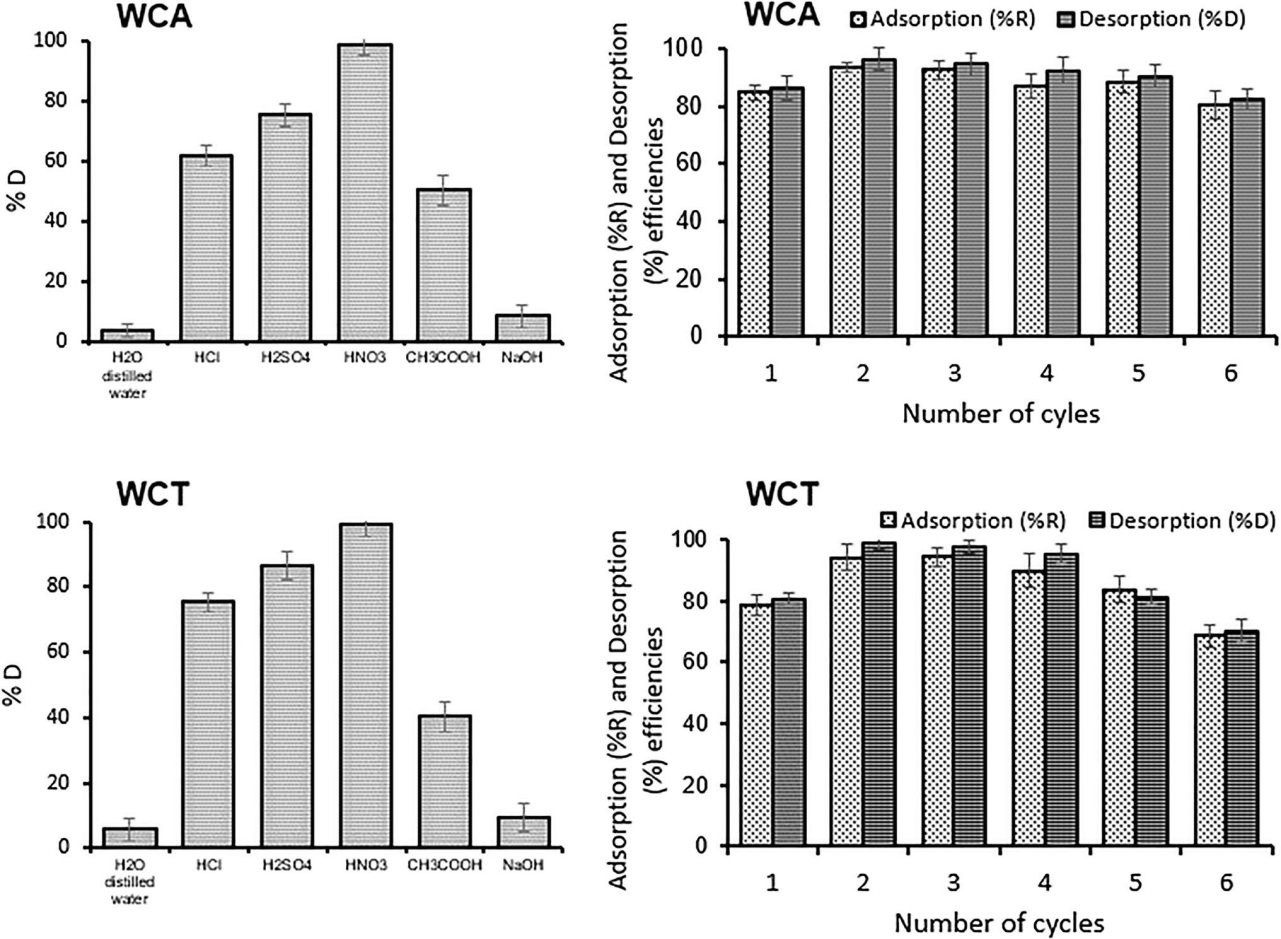
Pb(II) desorption and regeneration. [Left] desorption using five different solvents. [Right] cycles of sorption (%R) and desorption (%D) efficiencies for 0.1M HNO_3_ eluent

Figure 10 shows the results of the desorption and regeneration experiments of the biosorbents WCA and WCT. For both biosorbents five eluents have been used; three of them show high desorption percentages (%D > 60) of Pb(II), because they are acids that release proton H^+^ and replace by Pb(II) on the surface of biosorbents (Ezeonuegbu et al. 2021). The most efficient eluent is HNO_3_ with which Pb(II) was desorbed up to %D = 98.5 and 99.3 for WCT and WCA, respectively (Fig. 10, left). With this eluent, up to six sorption/desorption cycles were carried out. For both biosorbents, the recovery efficiency is high (> 80%) up to five cycles, after that the efficiency decreases significantly. It can also be noted that the sorption (%R) and desorption (%Des) efficiencies increase in the 2^nd^ cycle. This behavior is due to the fact that HNO_3_ can dissolve the organic parts of biosorbents, activating more Pb(II) adsorption sites. The decrease in %R when increasing the number of cycles was also reported by Elkhaleefa et al. (2021) and Tran et al. (2016).

## Conclusions

The interest in the development of low-cost biomaterials for the Pb removal from contaminated waters led to the investigation of Pb(II) sorption processes on *arabica*–coffee (WCA) and *theobroma*–cocoa (WCT) agro-industrial waste biosorbents. It has been determined zero charge point pH values (pH_PZC_) at 4.8 and 3.9 for WCA and WCT, respectively.

IR spectra before and after biosorption showed changes in the intensity and position of bands mainly associated with vibrational groups O-H, C-O and C-O-C.

SEM/EDS analyzes show cracked surfaces with heterogeneous plates. This morphology undergoes significant changes towards greater homogeneity after the Pb(II) sorption.

Both biosorbents practically reach the maximum percentage of adsorption (%R > 90) for a mass of 0.05 g (dose=2 mg⋅L^-1^) with a [Pb(II)] concentration C_0_ of 26.96 mg⋅L^-1^, at pH = 4.5 and sorption time t_sp_ = 120 min.

From the adsorption isotherms, adjustment to the Langmuir model, were derived the maximum adsorption capacities, q_max_, of 158.7 and 123.5 mg⋅g^-1^ for WCA and WCT, respectively.

The kinetics of the sorption processes are very well adjustment to the pseudo-2^nd^-order model. The corresponding parameters indicate that these are fast chemisorption processes, particularly for WCA (almost six times larger than for WCT).

Desorption-regeneration experiments show that HNO_3_ is the most efficient eluent, which Pb(II) are recovered efficiently, up to more than 98% (particularly in the 2^nd^ cycle). Both WCA and WCT biosorbents, can be re-used up to five times.

## Data Availability

If any researchers need original data of this manuscript, the authors agree to provide relevant information.
